# P-2298. Daily Single-strength vs. Thrice-weekly Double-strength Trimethoprim/sulfamethoxazole for *Pneumocystis jirovecii* Prophylaxis in Kidney Transplant Recipients: A Non-inferiority Randomized Controlled trial

**DOI:** 10.1093/ofid/ofae631.2451

**Published:** 2025-01-29

**Authors:** Wittawin Chantapoh, Surasak Kantachuvesiri, Napan Sutharattanapong, Jackrapong Bruminhent

**Affiliations:** Faculty of Medicine Ramathibodi Hospital, Mahidol University, Bangkok, Krung Thep, Thailand; Faculty of Medicine Ramathibody Hospital, Mahidol University, Bangkok, Krung Thep, Thailand; Faculty of Medicine Ramathibodi Hospital, Mahidol University, Bangkok, Krung Thep, Thailand; Faculty of Medicine Ramathibodi Hospital, Mahidol University, Bangkok, Krung Thep, Thailand

## Abstract

**Background:**

*Pneumocystis jirovecii* pneumonia (PCP) is commonly encountered among kidney transplant (KT) recipients. Either daily or thrice-weekly, single strength (SS) or double strength (DS) trimethoprim/sulfamethoxazole (TMP/SMX) is recommended after KT to prevent PCP. However, a direct comparison between these regimens in terms of PCP prevention is lacking. We assessed the efficacy of SS daily (DSS) versus DS thrice weekly (TDS) TMP/SMX prophylaxis for PCP within the first 6 months post-transplant in KT recipients.

**Methods:**

We conducted a single-center block of 4 randomized, open-label, non-inferiority, randomized controlled trial at a single transplant center between December 2022 and December 2023. All KT recipients 15 years and older were included. The single-strength group received TMP/SMX 80/400 mg daily after KT, while the double-strength group received two tablets of SS doses thrice weekly. PCP occurrence was observed for 6 months, and side effects of TMP/SMX and ease of drug consumption were monitored.

**Results:**

A total of 65 KT recipients were included. The participants had a mean age (SD) of 46 (11) years old, with the following proportion: male gender (58%), deceased donor KT (20%), anti-thymocyte globulin as induction therapy (17%), and CMV seropositivity (95%). There were 32 and 33 KT recipients assigned to the DSS and TDS groups, respectively. Baseline characteristics between the two groups were comparable. At 6 months after transplant, all patients remained free from PCP in both groups. There was a non-significant lower mean (SD) of white blood cell counts in the DSS group compared to the TDS group [6,235 (2,271) vs.7,052 (2,464) cells/mm^3^, p = 0.23], respectively. In parallel, there was no significant difference in terms of hepatotoxicity or renal dysfunction among those groups. According to per-protocol analysis, 27 (96%) of the DSS group reported having convenient administration of the drug compared to 18 (100%) of the TDS group (p = 0.60).

**Conclusion:**

TMP/SMX appears to efficiently prevent PCP in the early period after KT. Both SS daily and DS thrice-weekly regimens seem to be comparable in terms of effectiveness, tolerability, and compliance. Further studies with larger samples are encouraged to support this result. TCTR20240329004.

**Disclosures:**

All Authors: No reported disclosures

